# Prognostic implication of erector spinae muscles in non‐small‐cell lung cancer patients treated with immuno‐oncology combinatorial chemotherapy

**DOI:** 10.1111/1759-7714.14142

**Published:** 2021-10-02

**Authors:** Taisuke Araki, Yoshiaki Kitaguchi, Yusuke Suzuki, Masamichi Komatsu, Kei Sonehara, Yosuke Wada, Kazunari Tateishi, Masayuki Hanaoka

**Affiliations:** ^1^ First Department of Internal Medicine Shinshu University School of Medicine Matsumoto City Japan

**Keywords:** carcinoma, erector spinae muscles, immunotherapy, non‐small‐cell lung cancer, nutrition assessment

## Abstract

**Background:**

The quantity of skeletal muscles has recently been reported to have prognostic value in patients with non‐small‐cell lung cancer (NSCLC) treated with second‐line immunotherapy. However, the prognostic role of skeletal muscle assessment in NSCLC patients undergoing first‐line immuno‐oncology (IO) combinatorial treatment (IO‐chemotherapy) has not been elucidated.

**Methods:**

We retrospectively reviewed 36 patients with NSCLC undergoing first‐line IO‐chemotherapy between April 2018 and June 2021 in our hospital. The cross‐sectional area of the erector spinae muscle (ESM_CSA_) was evaluated by manual tracing on computed tomography scans at the level of the 12th thoracic vertebra before initiating IO‐chemotherapy. To minimize deviation due to physique, the ESM_CSA_ was adjusted by body surface area (BSA) (ESM_CSA_ to BSA ratio: ESM_CSA_/BSA). A survival time analysis was performed using the Kaplan–Meier method and log‐rank test. A multivariate analysis with Cox proportional hazards model was conducted to investigate the prognostic value of the ESM_CSA_/BSA and inflammatory and nutritional indices.

**Results:**

The median progression‐free survival (PFS) and overall survival (OS) were 6.5 and 16.6 months, respectively. Intergroup comparison by the log‐rank test revealed that there was no significant difference in the median PFS, but the median OS was significantly long in the high ESM_CSA_/BSA (>19 cm^2/^m^2^) (high ESM_CSA_/BSA group, *p* = 0.0373). The multivariate analysis showed that ESM_CSA_/BSA was an independent prognostic factor for OS (hazard ratio 0.79, *p* = 0.044).

**Conclusions:**

The results of this study indicate that the pretreatment ESM_CSA_/BSA may be a potential prognostic factor in NSCLC patients receiving first‐line IO‐chemotherapy.

## Graphical abstract

In non‐small‐cell lung cancer patients undergoing first‐line immuno‐oncology combinatorial treatment, pretreatment erector spinae muscle/body surface area (ESM_CSA_/BSA) was associated with survival outcome. The lower ESM_CSA_/BSA group had significantly shorter overall survival. This finding was validated by the multivariate analysis performed in this study.

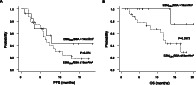

## INTRODUCTION

Immuno‐oncology (IO) combinatorial treatment (IO‐chemotherapy), which combines platinum doublet chemotherapy with immune checkpoint inhibitors (ICIs), such as anti‐programmed cell death‐1 (PD‐1)/programmed death ligand‐1 (PD‐L1) antibody and anti‐cytotoxic T‐lymphocyte antigen‐4 antibody, is one of the optimal first‐line treatment strategies for advanced or recurrent non‐small‐cell lung cancer (NSCLC) patients with negative or unknown status of oncogenic driver mutations. The concomitant use of ICIs with cytotoxic chemotherapy agents induces upregulation of antitumor immunity, resulting in synergistic therapeutic effects.^1^ Specifically, several phase III clinical trials demonstrated that IO‐chemotherapy is associated with better survival outcomes and treatment responses compared to conventional cytotoxic chemotherapy regimens.^2^, ^3^, ^4^, ^5^, ^6^

In patients with advanced NSCLC, potential nutritional status, physical activity represented by performance status (PS), and serum albumin levels are well‐known prognostic factors.^7^, ^8^, ^9^ Exhaustion due to tumorigenic systemic inflammation and toxicity of anticancer agents could accelerate malnutrition, leading to cancer cachexia.^10^ Numerous studies have demonstrated that inflammation‐ and nutrition‐associated indices, which consist of hematological and/or biochemical parameters, have prognostic potential in patients with advanced NSCLC. It has also been reported that a radiological assessment of the quantity and quality of skeletal muscles and its time‐dependent changes are also associated with prognosis in patients with advanced NSCLC.^11^, ^12^, ^13^, ^14^, ^15^ Antigravity muscles, which are involved in posture maintenance, are considered to better reflect the quality and quantity of physical activity compared to other skeletal muscles.^16^ Previous studies have shown that a skeletal muscle index calculated using measurements at the level of the third lumbar vertebra on computed tomography (CT) scans were significantly associated with progression‐free survival (PFS) and overall survival (OS) outcomes in NSCLC patients treated with ICI monotherapy.^12^, ^13^, ^14^, ^15^

The erector spinae muscles (ESMs) are included in the imaging level of a chest CT scan and can be quantitatively evaluated in mediastinal settings. In patients with advanced lung cancer, chest CT scans are routinely performed to evaluate tumor staging and treatment response to chemotherapy, which allows for a time series evaluation of ESMs. In recent years, the quantity of ESMs has been reported as a prognostic factor in patients with chronic obstructive pulmonary disease,^17^ idiopathic pulmonary fibrosis,^18^ pulmonary nontuberculous mycobacteria,^19^ and community‐acquired pneumonia.^20^ However, to the best of our knowledge, the prognostic value of ESMs has not been investigated in patients with advanced NSCLC who were treated with first‐line IO‐chemotherapy. Therefore, we hypothesized that in patients with advanced NSCLC treated with first‐line IO‐chemotherapy the quantity of ESMs before IO‐chemotherapy could be associated with their prognosis. The aim of this study was to examine the predictive and prognostic value of ESMs in patients with NSCLC undergoing first‐line IO‐chemotherapy.

## METHODS

### Patients and settings

The study was conducted retrospectively at a single institution. All methods used in this study were performed in accordance with the amended Declaration of Helsinki. The Institutional Review Board of Shinshu University School of Medicine approved the study (approval number: 4772) and waived the need for informed consent owing to its retrospective design. Instead, an opt‐out document for this study was posted on the website of Shinshu University School of Medicine. The data for analysis were collected from electrical medical records. We extracted information from patients who were histologically diagnosed with NSCLC between April 2018 and June 2021. All patients met the following criteria: having advanced or recurrent NSCLC and receiving at least one cycle of ICIs in combination with platinum‐based chemotherapy as first‐line chemotherapy.

### Data collection

Data on patient characteristics included age, sex, smoking history, performance status (PS) evaluated by the Eastern Cooperative Oncology Group scale, body mass index (BMI), body surface area (BSA), histology findings, tumor stage assessed by tumor‐node‐metastasis eighth stage classification,^21^ PD‐L1 status, and oncogenic driver mutations. PD‐L1 status was evaluated in archival or newly obtained tumor samples by means of immunohistochemical analysis with the use of the 22C3 antibody. The baseline data before initiating IO‐chemotherapy included white blood cell counts and its fractions, and serum albumin levels. Based on these parameters, the neutrophil‐to‐lymphocyte ratio (NLR), prognostic nutrition index (PNI), and advanced lung cancer inflammation index (ALI) were generated. The NLR was calculated as the ratio of absolute neutrophil count (ANC) to lymphocyte count (ALC), and patients were classified as having <5 or ≥5. The PNI score was calculated as 10× albumin + 0.005 × ALC, and patients were classified as having ≤40 or >40. The ALI score was calculated as (BMI × albumin)/NLR, with patients classified as having <18 or ≥18. The cutoff values for NLR, PNI, and ALI were determined according to the respective seminal reports.^22^, ^23^, ^24^

### Clinical course

Data on first‐line IO‐chemotherapy, adverse events (evaluated by the Common Terminology Criteria for Adverse Events version 5.0),^25^ treatment response (evaluated by the Response Evaluation Criteria in Solid Tumors version 1.1),^26^ and the survival time were collected. PFS for first‐line IO‐chemotherapy was defined as the period from the initiation of IO‐chemotherapy until death or disease progression. OS was defined as the period from the initiation of IO‐chemotherapy until either a fatal event or censored observation.

### Quantitative analysis of the ESM area

All patients underwent chest CT during an inspiratory breath‐hold in the supine position with a 64‐row multidetector CT scanner (LightSpeed VCT, GE Healthcare). The CT scanner settings were 120 kV tube voltage, variable tube current, 64 × 0.625 mm collimation, and a 0.4‐s rotation time. Image reconstruction was performed with the standard algorithm for the mediastinum and a slice thickness of 1.25 mm. Following a method described in previous studies,^19^, ^27^ a quantitative analysis of the ESM area was performed using a commercially available workstation (EV Insite, PSP Co.) before initiating IO‐chemotherapy. In summary, the position of the analysis slice was determined at the level of the lower margin of the 12th thoracic vertebra, and the limb of the left and right ESMs area was bordered by manual tracing on a single axial image. The cross‐sectional areas (CSAs) of both ESMs (ESM_CSA_) were automatically calculated, and the ESM_CSA_ was presented as the sum of the area of right and left ESMs. The ESM_CSA_ was adjusted by the BSA, and the ratio of the ESM_CSA_ to the BSA was employed in the analysis as ESM_CSA_/BSA. The chest CT images were reviewed by two expert pulmonologists (T.A. and Y.K., with 10 and 22 years of experience, respectively) by consensus. The correlation of the physique index with the ESM_CSA_ was also evaluated.

### Statistical analysis

Spearman's rank correlation coefficient was used to investigate the correlation between the ESM_CSA_ and physique variables. A receiver operating characteristic (ROC) curve was constructed using the pretreatment ESM_CSA_/BSA as the test variables and death events (death or survival and censored events) as the state variables. The optimal cutoff value for the ESM_CSA_/BSA was assessed by calculating the area under the ROC curves for predicting death events to perform a comparative analysis. A Kaplan–Meier analysis was performed to plot the PFS and OS curves, and the log‐rank test was used for intergroup comparisons of PFS and OS. A Cox proportional hazards model was used to identify the prognostic factors for PFS and OS. Statistically significant variables were determined using the univariate model and clinically important variables were further analyzed using the multivariate analysis. All statistical analyses were performed using EZR (Saitama Medical Center, Jichi Medical University, Saitama, Japan), a graphical user interface for R (The R Foundation for Statistical Computing). Statistical significance was set at *p* < 0.05.^28^

## RESULTS

### Patient characteristics

During the study period, data on 38 patients with advanced or recurrent NSCLC treated with first‐line IO‐chemotherapy were extracted. One patient was excluded due to missing CT scan data and another patient was excluded due to receipt of IO‐chemotherapy as second‐line chemotherapy. A total of 36 patients were included in the analysis. The patient characteristics are presented in Table 1. The cohort comprised 31 (86.1%) male and five (13.9%) female patients, with a median age of 68.5 years (range 49–79 years).

**TABLE 1:** Patient characteristics (*n* = 36)

	*n*	%
Median age, year (range)	68.5 (49–79)	
Male sex	31	86.1
Smoking status		
Yes/no	33/3	91.7/8.3
BMI, kg/m^2^	21.6 ± 3.4	
BSA, m^2^	1.64 ± 0.16	
ECOG‐PS		
0,1	28	77.8
2,3	8	22.2
Histology		
Adeno	24	66.7
Squamous	6	16.7
NSCLC‐NOS	5	13.9
Large cell	1	2.8
Tumor stage		
III	3	8.3
IVA	11	30.1
IVB	21	58.3
Post. Ope	1	2.8
PD‐L1 status		
<1%	7	19.4
≥1%	23	63.9
Unknown	6	16.7
Presence of driver mutation	4	11.1
Clinical parameters		
NLR	4.9 ± 3.3^a^	
<5/≥5	22/14	61.1/38.9
PNI	41.2 ± 6.0^a^	
≤40/>40	15/21	41.7/58.3
ALI	20.9 ± 14.5^a^	
<18/≥18	17/19	47.2/52.8
ESM_CSA_, cm^2^	29.6 ± 7.3^a^	
ESM_CSA_/BSA, cm^2^/m^2^	17.9 ± 3.5^a^	

Abbreviations: ALI, advanced lung cancer inflammation index; BMI, body mass index; BSA, body surface area; CSA, cross‐sectional area; ECOG‐PS, Eastern Cooperative Oncology Group performance status; ESM, erector spinae muscles; NLR, neutrophil to lymphocyte ratio; NOS, not otherwise specified; NSCLC, non‐small cell lung cancer; PD‐L1, programmed cell death ligand 1; PNI, prognostic nutrition index.

^a^ Mean ± standard deviation.

More than 80% of patients had nonsquamous histology, including 24 (66.7%) adenocarcinoma, five (13.9%) NSCLC not otherwise specified, and one (2.8%) large cell carcinoma. PD‐L1 status was available in 30 (83.3%) patients, of which 23 (63.9%) had positive expression (≥1%), and four (11.1%) carried the oncogenic driver mutation. The baseline ESM_CSA_ was 29.6 ± 7.3 cm^2^. Since a significant difference was found between the ESM_CSA_ and BSA, the ESM_CSA_ was adjusted by the BSA (*r* = 0.599, *p* < 0.001; Supporting Information Table S1) (ESM_CSA_ to BSA ratio: ESM_CSA_/BSA). The baseline ESM_CSA_/BSA was 17.9 ± 3.5 cm^2^/m^2^.

### Adverse events

All the patients exhibited adverse events (AEs). Neutropenia was the most frequent grade 3 AE (30.6%), followed by adrenal insufficiency and bacterial pneumonia (8.3% each), excluding the hematological and gastrointestinal toxicities (Table 2).

**TABLE 2:** Adverse events for IO‐chemotherapy (*n* = 36)

Variables	Any grade *n* (%)	Grade3≤ *n* (%)
Neutropenia	19 (52.3)	11 (30.6)
Anemia	21 (58.3)	1 (2.8)
Thrombocytopenia	14 (38.9)	3 (8.3)
Nausea	24 (66.7)	8 (22.2)
Diarrhea	5 (13.9)	0 (0)
Rash	14 (38.9)	0 (0)
Liver dysfunction	9 (25)	1 (2.8)
Renal dysfunction	8 (22.2)	0 (0)
Peripheral neuropathy	7 (19.4)	1 (2.8)
Thyroid dysfunction	6 (16.7)	0 (0)
Interstitial lung disease	4 (11.1)	0 (0)
Adrenal insufficiency	5 (13.9)	3 (8.3)
Bacterial pneumonia	3 (8.3)	3 (8.3)
Bacterial infection	3 (8.3)	1 (2.8)

### Treatment response and survival time analysis

**FIGURE 1:**
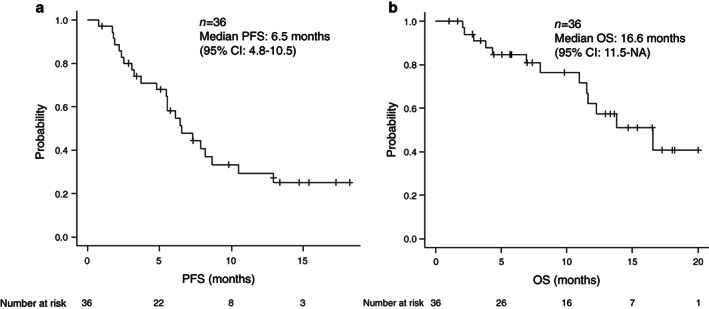
The Kaplan–Meier curve for the entire cohort (*n* = 36) is presented. The median progression‐free survival (PFS) was 6.5 months (a) and median overall survival was 16.6 months (b)

**FIGURE 2:**
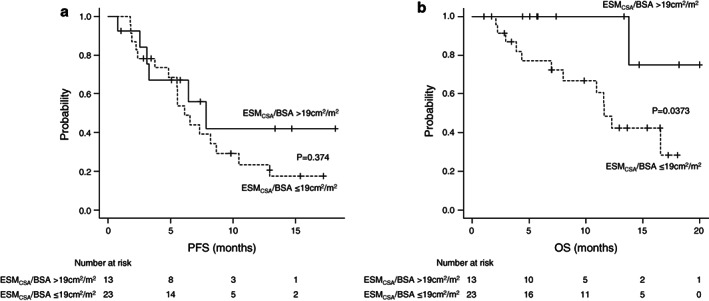
Survival time comparison according to the ESM_CSA_/BSA is shown. The median PFS tended to be long in the high ESM_CSA_/BSA group (>19 cm^2^/m^2^, solid line), with no statistical significance (a). The median OS was significantly long in the high ESM_CSA_/BSA group (b). BSA, body surface area; CSA, cross‐sectional area; ESM, erector spinae muscle; OS, overall survival; PFS, progression free survival

The treatment regimen, efficacy, and clinical course are presented in Table 3. Twenty‐five patients (69.4%) received platinum‐doublet chemotherapy with pembrolizumab, six (16.7%) received platinum‐doublet chemotherapy with atezolizumab, and five (13.9%) received platinum‐doublet chemotherapy with nivolumab and ipilimumab as first‐line IO‐chemotherapy. The objective response rate and disease control rate for first‐line IO‐chemotherapy were 58.3% (95% confidence interval [CI] 40.8–74.5) and 86.1% (95% CI 70.5–95.3), respectively. During the observational period, 23 patients (63.9%) showed disease progression and 13 (36.1%) patients died. The median PFS and OS in the entire cohort were 6.5 months (95% CI 4.8–10.5) and 16.6 months (95% CI 11.5 to not applicable [NA]), respectively (Figure 1). Table 4 presents the results of the comparative analysis of PFS and OS according to baseline patient characteristics, clinical parameters, and ESM_CSA_/BSA. The median OS was long in the group with a high ESM_CSA_/BSA (>19 cm^2^/m^2^) (≤19 vs. >19 cm^2/^m^2^, 11.6 months vs. NA, *p* = 0.0373) (Figure 2). The results of the univariate analysis for PFS and OS are presented in Table 5. There were no significant prognostic factors associated with PFS. The baseline ESM_CSA_/BSA was found to be a significant prognostic factor for OS (hazard ratio [HR] 0.78, 95% CI 0.62–0.98, *p* = 0.041). The multivariate analysis using the Cox proportional hazards model demonstrated that the baseline ESM_CSA_/BSA was an independent prognostic factor for OS (HR 0.79, 95% CI 0.62–0.99, *p* = 0.0442) (Table 6).

**TABLE 3:** Clinical course (*n* = 36)

	*n*	%
Treatment regimen		
Pembrolizumab + chemo	25	69.4
Atezolizumab + chemo	6	16.7
Nivo + Ipi + chemo	5	13.9
Treatment response		
ORR (%), 95% CI	58.3	40.8–74.5
DCR (%), 95% CI	86.1	70.5–95.3
No. of progression events	23	63.9
No. of mortality events	13	36.1

Abbreviations: CI, confidence interval; DCR, disease control rate; Ipi, ipilimumab; Nivo, nivolumab; ORR, objective response rate.

**TABLE 4:** Survival time comparison by log‐rank test (*n* = 36)

Variables	PFS		OS	
	Months	*p* value	Months	*p* value
Age	6.5 vs. 5.5	0.543	13.8 vs 16.6	0.254
≥70 (*n* = 19) vs. <70				
Sex	8.2 vs. 6.4	0.34	16.6 vs. 13.8	0.494
Male (*n* = 31) vs. female				
ECOG‐PS	5.6 vs. 6.5	0.558	NA vs. 16.6	0.634
2,3 (n = 8) vs. 0,1				
PD‐L1	7.3 vs. 8.2	0.997	NA vs. 13.8	0.865
≥1% (*n* = 23) vs. <1%				
Driver mutation	5.6 vs. 7.3	0.928	NA vs. 13.8	0.318
Yes (*n* = 4) vs. no				
NLR	8.6 vs. 6.4	0.494	12.3 vs. 16.6	0.998
≥5 (*n* = 14) vs. <5				
PNI	5.6 vs. 7.3	0.982	16.6 vs. 13.8	0.97
≤40 (*n* = 15) vs. >40				
ALI	6.5 vs. 6.4	0.452	12.3 vs. 16.6	0.926
<18 (*n* = 17) vs. ≥18				
ESM_CSA_/BSA	6.1 vs. 7.9	0.374	11.6 vs. NA	0.0373
≤19 cm^2^/m^2^ (*n* = 23) vs. >19 cm^2^/m^2^				

Abbreviations: ALI, advanced lung cancer inflammation index; BSA, body surface area; CSA, cross‐sectional area; ECOG‐PS, Eastern Cooperative Oncology Group performance status; ESM, erector spinae muscle; NA, not applicable; NLR, neutrophil‐to‐lymphocyte ratio; OS, overall survival; PD‐L1, programmed cell death ligand 1; PFS, progression‐free survival; PNI, prognostic nutrition index.

**TABLE 5:** Univariate analysis for PFS and OS (*n* = 36)

Variables	PFS		OS	
	HR (95% CI)	*p* value	HR (95% CI)	*p* value
Age	1.03 (0.97–1.1)	0.323	1.01 (0.93–1.09)	0.818
Male sex	1.64 (0.59–4.6)	0.346	0.59 (0.13–2.71)	0.499
ECOG‐PS 2,3	0.73 (0.25–2.14)	0.561	1.37 (0.37–5.03)	0.635
PD‐L1 of ≥1% or <1%	0.99 (0.36–2.79)	0.997	0.89 (0.24–3.37)	0.865
Presence of driver mutation	1.47 (0.43–4.95)	0.538	NA	NA
NLR	0.99 (0.85–1.16)	0.902	1.07 (0.89–1.28)	0.497
PNI	0.99 (0.93–1.06)	0.816	0.97 (0.88–1.07)	0.545
ALI	0.99 (0.97–1.03)	0.983	0.97 (0.93–1.02)	0.281
ESM_CSA_/BSA	0.91 (0.79–1.05)	0.201	0.78 (0.62–0.98)	0.041

Abbreviations: ALI, advanced lung cancer inflammation index; BSA, body surface area; CI, confidence interval; CSA, cross‐sectional area; ECOG‐PS, Eastern Cooperative Oncology Group performance status; ESM, erector spinae muscle; HR, hazard ratio; NA, not applicable; NLR, neutrophil‐to‐lymphocyte ratio; OS, overall survival; PD‐L1, programmed cell death ligand 1; PFS, progression‐free survival; PNI, prognostic nutrition index.

**TABLE 6:** Cox proportional hazards model for OS (*n* = 36)^a^

Variables	HR (95% CI)	*p* value
ECOG‐PS 2,3	1.43 (0.37–5.55)	0.605
ESM_CSA_/BSA	0.79 (0.62–0.99)	0.0442

Abbreviations: BSA, body surface area; CI, confidence interval; CSA, cross‐sectional area; ESM, erector spinae muscle; HR, hazard ratio; OS, overall survival; PS, performance status.

^a^ Including 13 mortality events.

## DISCUSSION

The present study was conducted to investigate pretreatment ESMs as prognostic markers in patients with advanced NSCLC receiving first‐line IO‐chemotherapy. The survival time analysis using log‐rank test showed that the group with a high ESM_CSA_/BSA had significantly long OS, and the univariate analysis revealed that the ESM_CSA_/BSA was significantly associated with poor OS. The baseline ESM_CSA_/BSA was identified as an independent prognostic factor for OS in the multivariate analysis using the Cox proportional hazards model. There were no significant prognostic values in the survival time analysis for the inflammation‐ and nutrition‐related indices evaluated in this study. To our knowledge, this is the first study to demonstrate the prognostic relevance of ESM_CSA_/BSA in patients with NSCLC undergoing first‐line IO‐chemotherapy.

In patients with advanced cancer, it is well known that tumorigenic systemic inflammation leads to muscle proteolysis, lipolysis, and appetite suppression by involving various inflammatory cytokines.^10^ In turn, this leads to further skeletal muscle loss and undernutrition, resulting in a state known as cachexia. In patients with NSCLC treated with second‐line ICI therapy, a poor PS is a well‐known adverse prognostic factor.^29^ In recent years, several clinical parameters derived from hematological and/or biochemical laboratory test results have been explored to evaluate the predictive and prognostic value of second‐line ICI therapy in NSCLC patients in real‐world settings. The NLR,^30^ PNI score,^31^ and ALI score, ^32^ which were investigated in this study, have been well described as potential prognostic factors in advanced NSCLC undergoing second‐line ICI therapy. However, the prognostic significance of inflammatory and nutritional factors in NSCLC patients undergoing first‐line IO‐chemotherapy is limited owing to the novel nature of its populations. Morimoto et al. recently reported that in a multicenter retrospective study of NSCLC patients undergoing IO‐chemotherapy, patients with cachexia had significantly shorter PFS than those without cachexia. OS tended to be shorter in the cachexia group than in those without, with no statistical significance in that study.^33^ This suggests that neoplastic inflammation and malnutrition have prognostic relevance even in patients with NSCLC undergoing IO‐chemotherapy regimens.

The skeletal muscle index evaluated by CT scans at the level of the third lumbar vertebra has been well investigated as an objective nutrition‐related indicator. Takada et al. reported that the skeletal muscle index was an independent prognostic factor for response rate and survival time outcomes in patients with advanced NSCLC treated with second‐line anti‐PD‐1 antibody therapy.^14^ Similarly, in their multicenter observational study, Cortellini et al. also reported that a lower skeletal muscle index was significantly associated with shorter OS time in advanced NSCLC patients treated with anti PD‐1/PD‐L1 antibody therapy.^15^ It was noted that these previous studies included patients who received second‐line ICI therapy and did not concurrently investigate nutritional and inflammatory indices other than muscle indices. In addition, skeletal muscle assessment was based on the level of the third lumbar vertebra, and the ESMs have not been validated. ESMs is a parameter that has shown its prognostic value in several respiratory diseases. ESMs at the level of the 12th thoracic vertebra have the advantage of providing an easy and comprehensive evaluation on chest CT scans. The quantity of ESMs at the level of the 12th thoracic vertebra has been reported to be correlated with skeletal muscles at the level of fourth lumber vertebra,^34^ suggesting that it may have diagnostic value identical to that of the lumbar level muscles.

The results of the present study showed that a lower baseline ESM_CSA_/BSA tended to have a shorter PFS without statistical significance. Meanwhile, a lower baseline ESM_CSA_/BSA was considered an independent adverse prognostic factor for OS. These findings suggest that the baseline ESM_CSA_/BSA may be a potential prognostic marker rather than a predictive marker in NSCLC patients undergoing first‐line IO‐chemotherapy. In the investigated inflammatory and nutritional indices, none of these was associated with OS in the univariate analysis. The reason for this result is unclear, but our consideration is that clinical parameters consisting of hematological and biochemical tests, while better than single test values, might vary widely depending on the patient's background factors. In this respect, the baseline ESM_CSA_/BSA, which also varies with age and physique, may have a relatively good prognostic ability since it is adjusted by the physique variable.

The present study has several limitations. First, it was a retrospective study conducted at a single institution with a relatively small sample size. These factors could potentially cause a patient selection bias. Therefore, the interpretation of our results should be cautious, and a prospective study with a large sample size is desirable in the future. Second, this study lacked clinical details of second‐line chemotherapy after IO‐chemotherapy, which could affect the survival time. Third, the ESM_CSA_ was measured by manual tracing at specific prespecified CT levels. Previous studies have employed auto‐tracing using a specific analysis software. It is uncertain which method is superior as a tracing method; however, manual tracing can be implemented using software installed in existing electronic medical records systems and might be cost‐effective.

Despite the above limitations, this is the first study to suggest the prognostic implications of ESM_CSA_/BSA assessment in patients with NSCLC receiving first‐line IO‐chemotherapy. In our study cohort, the ESM_CSA_/BSA seemed to have a superior potential prognostic ability compared to the other nutritional and inflammatory indices. Since this study was conducted in a small number of patients and the observation period was short, further follow‐up and case accumulation should be attempted in the future to verify the prognostic value of the ESM_CSA_/BSA.

## CONFLICT OF INTEREST

The authors declare no competing interests.

## AUTHOR CONTRIBUTIONS

The study was originally conceived by Y.K. and T.A., and Y.S. handled the data collection and analysis. All the authors contributed to the conception of the study design. The first draft of the manuscript was written by T.A., and all the authors referred to and agreed to the final manuscript.

## ETHICS APPROVAL

The present study was approved by the Institutional Review Board of Shinshu University School of Medicine (approval no. 4772), and the study was performed in accordance with the ethical standards of the amended Declaration of Helsinki.

## Supporting information


**Table S1**. Presenting the results of Spearman's rank correlation coefficient. Among the parameters examined, there was the strongest positive correlation between ESM_CSA_ and BSA.
