# Supplementary material for: Prognostic implication of erector spinae muscles in non‐small‐cell lung cancer patients treated with immuno‐oncology combinatorial chemotherapy
Source: Thorac Cancer. 2021 Oct 2;12(21):2857–64. doi: 10.1111/1759-7714.14142 (PMC8563148; doi:10.1111/1759-7714.14142)
Supplement: Supplementary file 1 — Table S1. Presenting the results of Spearman's rank correlation coefficient. Among the parameters examined, there was the strongest positive correlation between ESMCSA and BSA. [file TCA-12-2857-s001.docx]

| **Supplemental Table S1** |  | |
| --- | --- | --- |
| Variable | ***r*** | **P value *** |
| Height | 0.368 | 0.0273 |
| Body weight | 0.596 | 0.000169 |
| BSA | 0.599 | 0.000151 |
| Albumin | -0.0413 | 0.811 |

* Spearman's rank correlation coefficient

Abbreviations: BSA, body surface area.

Supporting information

Table S1. Presenting the results of Spearman's rank correlation coefficient. Among the parameters examined, there was the strongest positive correlation between ESM_CSA_ and BSA.
